# Determining Haemodynamic Wall Shear Stress in the Rabbit Aorta *In Vivo* Using Contrast-Enhanced Ultrasound Image Velocimetry

**DOI:** 10.1007/s10439-020-02484-2

**Published:** 2020-03-04

**Authors:** K. Riemer, E. M. Rowland, C. H. Leow, M. X. Tang, P. D. Weinberg

**Affiliations:** Department of Bioengineering, Imperial College London, Prince Consort Road, London, SW7 2AZ UK

**Keywords:** Vector flow imaging, Wall tracking, UIV, Echo PIV, WSS, Atherosclerosis, Coronary heart disease, Hemodynamics

## Abstract

Abnormal blood flow and wall shear stress (WSS) can cause and be caused by cardiovascular disease. To date, however, no standard method has been established for mapping WSS *in vivo*. Here we demonstrate wide-field assessment of WSS in the rabbit abdominal aorta using contrast-enhanced ultrasound image velocimetry (UIV). Flow and WSS measurements were made independent of beam angle, curvature or branching. Measurements were validated in an *in silico* model of the rabbit thoracic aorta with moving walls and pulsatile flow. Mean errors over a cardiac cycle for velocity and WSS were 0.34 and 1.69%, respectively. *In vivo* time average WSS in a straight segment of the suprarenal aorta correlated highly with simulations (PC = 0.99) with a mean deviation of 0.29 Pa or 5.16%. To assess fundamental plausibility of the measurement, UIV WSS was compared to an analytic approximation derived from the Poiseuille equation; the discrepancy was 17%. Mapping of WSS was also demonstrated in regions of arterial branching. High time average WSS (TAWSS_*xz*_ = 3.4 Pa) and oscillatory flow (OSI_*xz*_ = 0.3) were observed near the origin of conduit arteries. In conclusion, we have demonstrated that contrast-enhanced UIV is capable of measuring spatiotemporal variation in flow velocity, arterial wall location and hence WSS *in vivo* with high accuracy over a large field of view.

## Introduction

Hemodynamic wall shear stress (WSS) is the frictional force per unit area exerted by the flow of blood on the inner surface of blood vessels. It is the product of the near-wall velocity gradient and the viscosity of the blood. WSS is known to influence the normal physiology of the endothelial cells (EC) lining the wall and is also thought to be critical in the development of atherosclerosis in arteries, where spatial variation in WSS may explain the patchy distribution of disease.^15^,^20^,^21^,^24^,^32^ Under the assumption of steady, incompressible, laminar flow of a homogenous Newtonian fluid in a rigid cylindrical tube, WSS can be estimated using the Poiseuille equation. However, blood flow in large arteries is pulsatile, arteries branch, curve, twist, taper and translate, and their walls are viscoelastic. For vessels of this size, blood can be assumed a Newtonian fluid (i.e., its viscosity is independent of shear rate) but the WSS cannot be obtained analytically; it must be obtained numerically, which involves making assumptions about boundary conditions, or by measurement. The latter requires broad-view, angle-independent velocity measurement and wall tracking with high spatiotemporal resolution.

Contrast-enhanced ultrasound image velocimetry (UIV), also known as echo Particle Image Velocimetry (echoPIV), is based on 2D cross-correlation of speckle patterns in consecutive B-Mode images; it is capable of angle-independent measurement of the instantaneous velocity field in a plane.^13^,^17^,^22^,^30^,^35^ Physiological flow field velocity and hence shear rate can be resolved with high spatiotemporal resolution.^18^,^35^ With known location of the vessel-wall boundary, obtainable with high temporal resolution from the same images, the distribution of WSS in the vessel can be derived.^8^,^9^,^34^ Contrast agents greatly increase blood echogenicity and allow for better distinction between the vessel and the wall, facilitating WSS measurement. As the ultrasound frequency increases, filter techniques such as singular value decomposition (SVD)-based clutter filtering can be used in favor of nonlinear imaging schemes to further enhance the contrast to noise ratio.^6^

In this study, we demonstrate broad-view WSS measurements *in vivo* in the abdominal aorta of New Zealand White Rabbits using high-frame-rate, contrast-enhanced UIV independent of vessel number, beam angle, branching or curvature. First, we validate the vessel boundary tracking and velocity and WSS estimation in a realistic simulation of a pulsating straight vessel *in silico.* Next, we assess the vessel segmentation algorithm against manual segmentation of eight *in vivo* acquisitions by an experienced user. We demonstrate WSS measurement *in vivo* in a straight segment of the suprarenal abdominal aorta and compare the results to analytically derived and simulated solutions. Finally, we measure the WSS in two different regions of arterial branching—locations considered to be susceptible to disease formation—and qualitatively compare the distribution of instantaneous and time-average WSS to computational fluid dynamics (CFD) derived maps of WSS in the literature.

## Materials and Methods

### Flow and Ultrasound Simulations

**Figure 1:**
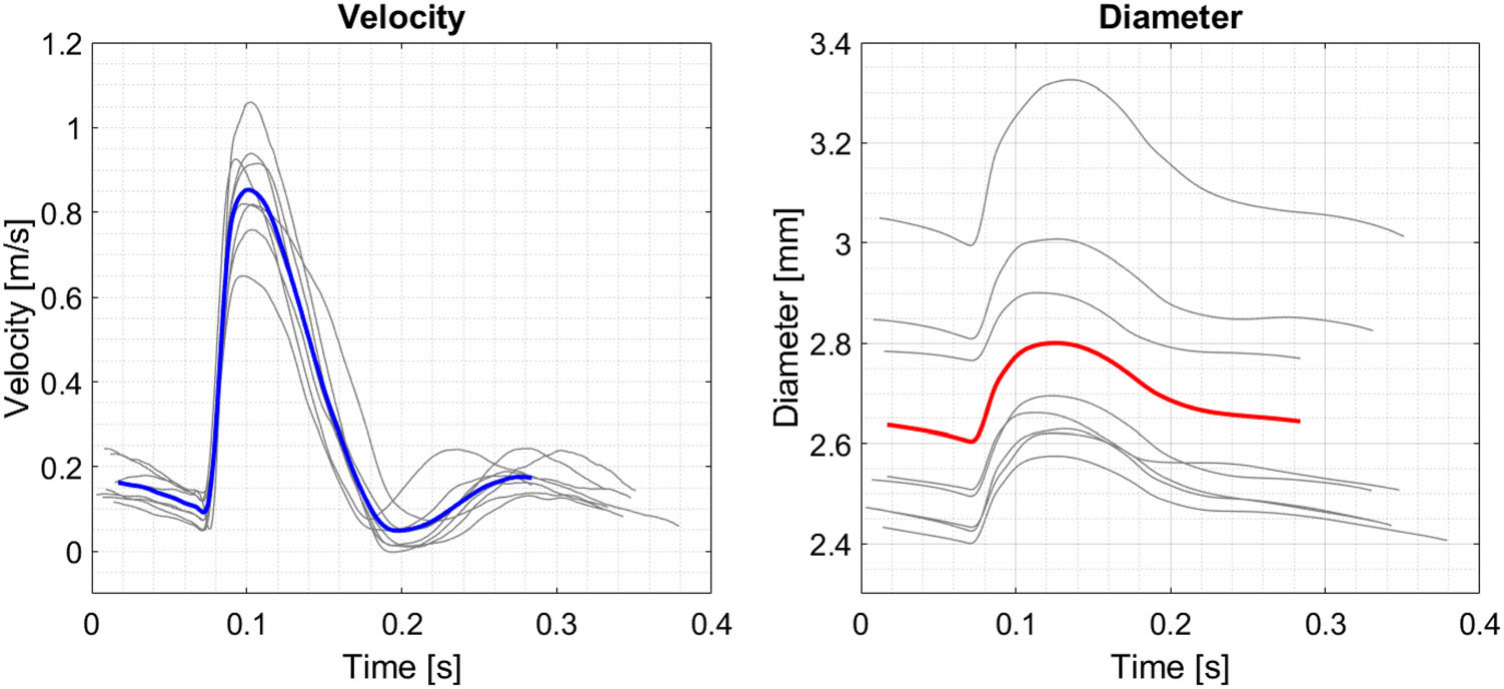
Single cardiac cycle velocity and vessel diameter waveforms from *in vivo* acquisitions in eight New Zealand White Rabbits. Individual waveforms were obtained in the thoracic aorta by averaging over 3 cycles. They were subsequently averaged to derive mean flow velocities (blue) and vessel displacements (red).

We first simulated the flow and then simulated the ultrasound acquisition of the flow. CFD simulations (StarCCM + v11) were conducted in a straight vessel with circular cross section and a diameter that changed over the cardiac cycle from 2.6 to 2.8 mm. This 8% wall displacement was based on the average diameter waveform for the rabbit descending thoracic aortas obtained from eight previously performed *in vivo* measurements (Fig. 1). Each measurement had been averaged over 3 cardiac cycles. Changes in vessel diameter were calculated using a 1D cross-correlation algorithm at 10 positions along the vessel wall. The inlet velocity waveforms were obtained from the same measurements (Fig. 1). Displacement of the vessel geometry and mesh generation conformed with the Courant criterion. In the simulation, vessel diameter was prescribed uniformly over the whole length of the 60 mm long vessel through a field function, using a mesh morpher. The wall was displaced incrementally prior to each time step according to the diameter waveform. Frequency and viscoelastic dependencies were ignored. The mean Reynolds number was Re_fb_ = 78. A constant pressure boundary condition was imposed at the vessel outlet. A cylindrical extension to the inlet with length (*L*) of 10 diameters (*D*) was added to generate the physiological conditions of a Womersley-like flow and a near fully-developed boundary layer. Blood was assumed to behave as a Newtonian fluid with density *ρ* = 1044.0 kg/m^3^ and viscosity *η* = 4.043 mPa·s. WSS was calculated for a single planar section through the central axis. To accommodate the high pulse repetition frequency (PRF) required for high frame rate vector flow imaging, the temporal resolution was set to 2.2e−4 per time step, equivalent to a PRF of 4500 Hz.

To generate simulated ultrasound images of the computed flow, a Verasonics 128 element L11-4v equivalent B-Mode imaging scheme was programmed in Field II.^10^,^23^ Scatters were randomly seeded in the vessel. The position $$\mathit{\mathbf{P_{{t_{n - 1} }}}} (\overset{\lower0.5em\hbox{$\smash{\scriptscriptstyle}$}} {\mathbf{x}} ,\overset{\lower0.5em\hbox{$\smash{\scriptscriptstyle}$}} {\mathbf{y}} ,\overset{\lower0.5em\hbox{$\smash{\scriptscriptstyle}$}} {\mathbf{z}} )$$ of each scatterer *n* in the flow region was displaced at each time step *t* by its respective CFD-derived displacement vector $$\overset{\lower0.5em\hbox{$\smash{\scriptscriptstyle}$}} {\mathbf{\mathit{V}}}_{{t_{n} }} (\overset{\lower0.5em\hbox{$\smash{\scriptscriptstyle}$}} {\mathbf{x}} ,\overset{\lower0.5em\hbox{$\smash{\scriptscriptstyle}$}} {\mathbf{y}} ,\overset{\lower0.5em\hbox{$\smash{\scriptscriptstyle}$}} {\mathbf{z}} )$$

$$\mathbf{\mathit{P_{{t_{n} }}}} (\overset{\lower0.5em\hbox{$\smash{\scriptscriptstyle}$}} {\mathbf{x}} ,\overset{\lower0.5em\hbox{$\smash{\scriptscriptstyle}$}} {\mathbf{y}} ,\overset{\lower0.5em\hbox{$\smash{\scriptscriptstyle}$}} {\mathbf{z}} ) = \mathbf{\mathit{P_{{t_{n - 1} }}}} (\overset{\lower0.5em\hbox{$\smash{\scriptscriptstyle}$}} {\mathbf{x}} ,\overset{\lower0.5em\hbox{$\smash{\scriptscriptstyle}$}} {\mathbf{y}} ,\overset{\lower0.5em\hbox{$\smash{\scriptscriptstyle}$}} {\mathbf{z}} ) + \overset{\lower0.5em\hbox{$\smash{\scriptscriptstyle}$}} {\mathbf{\mathit{V}}_{{t_{n} }}} (\overset{\lower0.5em\hbox{$\smash{\scriptscriptstyle}$}} {\mathbf{x}} ,\overset{\lower0.5em\hbox{$\smash{\scriptscriptstyle}$}} {\mathbf{y}} ,\overset{\lower0.5em\hbox{$\smash{\scriptscriptstyle}$}} {\mathbf{z}} ) \cdot \Delta T.$$

Tissue and flow were modeled with a Gaussian distribution around a mean intensity. The wall was modeled to generate a specular reflection. It was split into three layers, thereby mimicking the response of the intima, media, and adventitia.^29^ The wall thickness was uniform at *t =* 200 *μ*m and in accordance with *in vivo* data and previous results.^3^ To ensure a homogenous density of scatterers in the field of view of the transducer, particles were replaced based on their density in sub-regions. Each sub-region was a rectangle equivalent to 5% of the length × 5% of the width of the image; randomly placed scatterers were added to sub-regions in which their density was too low. This compensated for scatterers leaving at the outflows and not being replaced at the inflow The vessel was positioned with a beam-to-flow angle of 15°. Nonlinear effects of microbubbles were neglected. The transmit frequency was set to 8 MHz. Table 1 contains a complete list of parameters.

**Table 1:** Field II simulation setup and scatter properties.

Field II setup			
Centre frequency	6.25 MHz	Transmit frequency	8 MHz
No. of elements	128	Element pitch	3.00e−4 mm
Element width	2.7e−4 mm	Element height	5 mm
Sampling frequency	100 MHz	Elevational focus	18 mm
Number of sub-apertures	4	PRF	4500 Hz
Number of angles	3	Angle range	12°

Scatter properties	Mean	SD
Tissue	± 2.5	0.01
Inner wall layer (Intima)	0	1
Wall (Media)	± 2.5	0.01
Outer wall layer (Adventitia)	0	0.01
Microbubble	± 1.4	0.01

To validate the *in vivo* WSS measurements, a second CFD simulation in a straight vessel with circular geometry was performed. Wall displacement and velocity waveforms matched the measured values in the straight, unbranched segment of the descending thoracic aorta of a NZW rabbit. The temporal resolution was set to 1e−3 per time step. Velocity and WSS waveforms were directly compared between CFD simulation and *in vivo* UIV measurement.

### Ultrafast Plane Wave Imaging

A Verasonics Vantage 64/128 LE research ultrasound system with a L11-4v broadband probe was used. Ultrasound images were acquired with a PRF of 4500 Hz. Three plane waves with angles spanning 12° were transmitted per frame, giving a frame rate of 1500 fps. The transmit frequency was 8 MHz and the Mechanical Index was 0.1. Contrast agents were in-house manufactured decafluorobutane microbubbles.^17^,^28^ The radiofrequency data were beamformed using an in-house delay-and-sum (DAS) beam former and further analysis was performed in MATLAB (The Math-Works Inc., USA).

### Imaging the Rabbit Abdominal Aorta

**Figure 2:**
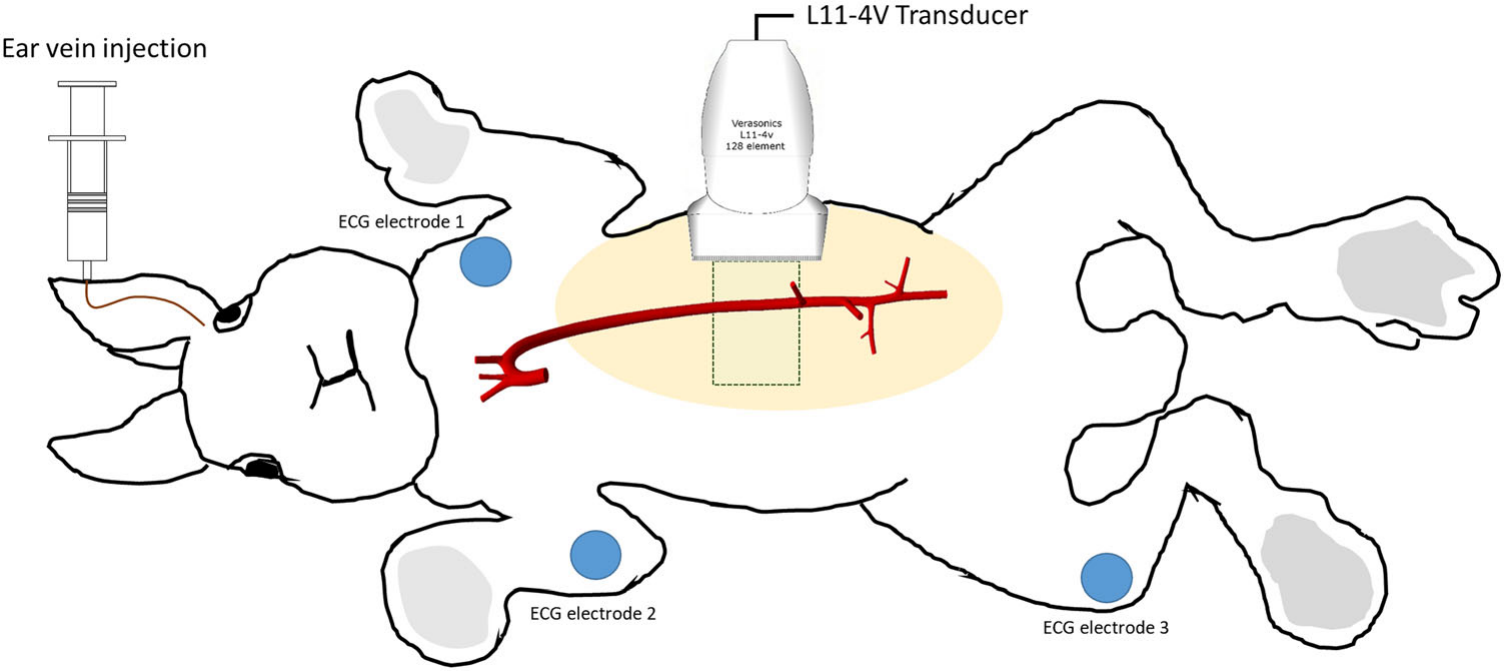
*In vivo* experimental setup and approximate transducer position, ECG electrode placement (blue) and area that needed to be shaved in order to perform the image acquisition (beige). The bottom rib and kidney were used as imaging landmarks. Ventilation was *via* tracheostomy. Illustration modified from Ref. 36.

Experiments were conducted on ten terminally anaesthetized specific-pathogen-free New Zealand White rabbits (HSDIF strain, 6–24 weeks; 2.2–3.3 kg; Envigo, UK). All experiments complied with the Animals (Scientific Procedures) Act 1986 and were approved by the Animal Welfare and Ethical Review Body of Imperial College London. Animals were housed individually in pens at 18–22 °C on a 12-h day–night cycle, and were given a standard laboratory diet and water *ad libitum*. Anesthesia was Acepromazine (0.5 mg/kg, *im*) followed by Hypnorm (fentanyl/fluanisone, 0.3 ml/kg initially plus 0.1 ml/kg every 45 min) and Midazolam (0.1 ml/kg, *iv* every 45 min). After tracheostomy, animals were ventilated at 40 breaths per minute. Body temperature was maintained with a heating mat and monitored with a rectal probe. An ECG signal was recorded synchronously with the ultrasound. Microbubbles were injected as a bolus (< 25 *μ*l/kg, iv). To perform the imaging, animals were placed supine, the fur around the image region was removed and ultrasound gel was applied (Fig. 2).

### Ultrasound Image Velocimetry Analysis

The UIV algorithm used in this study has previously been published.^17^,^18^ The basic principle is to calculate the displacement vector of all microbubbles in two consecutive B-Mode images separated by a known time delay in order to determine their velocity. Images were divided into interrogation windows (32 × 32 px) and a displacement vector for each patch was determined. The area of each interrogation window was iteratively decreased by half three times, the previous displacement being used as an initial estimate of displacement for its smaller successor; this procedure increased spatial resolution whilst reducing error and computational cost. In addition, a multi-grid window deformation,^27^ spurious vector elimination and sub-pixel displacement detection through Gaussian peak fitting were incorporated to further improve tracking accuracy.^22^ To minimize the effect of motion blur of the fast-flowing microbubbles in coherent compounded images, incoherent ensemble-correlation was performed.^18^ Each low resolution image of a multi-plane wave acquisition was cross-correlated with its respective consecutive image in time. Subsequently, an average correlation of all low resolution image pairs was used to determine the displacement field.

To improve the contrast to tissue ratio (CTR), a singular value decomposition (SVD) clutter filter was applied to each stack of low resolution images prior to the UIV analysis.^6^ This was done both for the simulated and experimental data. SVD factorizes the image data into a weighted, ordered sum of separable matrices of singular vectors and values. Due to their different spatiotemporal characteristics, it is assumed that tissue, blood and noise are represented by different sets of singular vectors. Reconstructing the signal using only singular vectors attributed to the flow highly increases the CTR.^6^ The selection of the correct singular values was based on the spatial similarity of the U-vectors of the SV decomposition.^2^ Similarly, post-analysis the displacement vector field was filtered using a proper orthogonal decomposition (POD).^11^ POD factorizes the flow field into a series of linear modes representing the energy in the flow. Modes with eigenvalues near zero correspond to low energy noise. Discarding low energy modes highly decreases spatial and temporal fluctuations and improves gradient estimation for the shear stress measurement.

### Vessel-Wall Segmentation

The abdominal aorta of the rabbit is often concealed by other vessels and signal echoes spread into adjacent regions beyond their true location. To accurately track the vessel-wall boundary position and orientation, a series of tracking and image augmentation steps were performed. First, the SVD clutter filter was used to decompose the acquisition into two datasets containing only tissue or only blood signals. A moving-window average was applied to each component. Images were normalized by their maximum and subtracted from each other, resulting in a high distinguishability between blood flow and vessel wall. Next, a sparse-field method (SFM) was applied to dynamically track the vessel wall in the augmented image, initialized by a manually placed mask in the first image.^33^ The SFM efficiently reduces the complexity of iterative active level-set contour models by only solving for the signed distance function near the zero level set.^16^ Last, because clutter-induced echoes were present in some parts of the vessel but not others, directional peak fitting (DPF) was applied. DPF defines the wall position to be the half-maximum point of the peak created by the vessel wall’s specular reflection closest to the lumen, effectively expanding the SFM mask, using it as a starting point and moving outwards. The vessel-wall boundary positions and orientations were automatically categorized as upper (anterior boundary) and lower (posterior boundary) relative to the transducer position. In cases of branching, vessels were further automatically divided into primary and secondary boundaries. Each boundary was individually filtered using a Savitzky–Golay filter to reduce the influence of noise and location errors. To assess the differences between ground truth *GT* and the proposed segmentation algorithm *M*, the Dice similarity coefficient (DSC) and the mean absolute distance between the walls (MADW) were calculated

$${\text{MADW}} = \frac{1}{n}\mathop \sum \limits_{i = 1}^{\text{np}} \left| {d(i,M,{\text{GT}})} \right|,$$

where np denotes the number of points of the contour of GT and *M*. The signed distance function between points from GT and the closest point in *M* is described as *d*.

### Wall Shear Stress and Oscillatory Shear Index

Since the vessels of interest were larger than 0.1 mm, the viscosity of blood was assumed to be constant.^7^ To calculate the WSR, every pixel in the image coordinate system **x**(x,y) was described by a velocity component **u**(u,v). The spatial distribution of WSS *τ*_w_ with a no-slip condition imposed on the wall is as follows 4,18

$$\tau_{\text{w}} = \mu_{\text{b}} \dot{\varepsilon }_{12}^{'}$$

where *μ*_b_ is the dynamic viscosity of blood at high shear rates and $\dot{\varepsilon }_{12}^{'}$ is the fluid strain rate tensor component oriented tangential to the wall and 12 describe the index of the strain rate tensor. To obtain $\dot{\varepsilon }_{12}^{'}$, the image-oriented strain rate tensor components $\dot{\varepsilon }_{ij}$

$$\dot{\varepsilon }_{ij} = \left[ {\frac{{\delta u_{i} }}{{\delta x_{j} }} + \frac{{\delta u_{j} }}{{\delta x_{i} }}} \right]$$

must be transformed using a transformation matrix *a*_*ij*_ at each pixel location of the vessel-wall boundary by the angle between the wall-oriented and image-oriented coordinate systems.

$$a_{ij} = \left[ {\begin{array}{*{20}c} {\cos \theta } & {\sin \theta } \\ { - \sin \theta } & {\cos \theta } \\ \end{array} } \right],$$

where *ij* describe the index of the strain rate tensor. Finally, the image-oriented strain according to second-rank tensors transformation is defined

$$\dot{\varepsilon }_{mn}^{'} = \mathop \sum \limits_{i = 1}^{2} \mathop \sum \limits_{j = 1}^{2} a_{mi} a_{nj} \dot{\varepsilon }_{ij} ,$$

where *m* and *n* are the pixel coordinates of the image.

The shear rate was estimated from the two closest points to the wall of a third order Savitzky–Golay filtered velocity profile over the vessel radius. The relative filter length was $n_{\text{s}} = \frac{n}{D} = 0.4,$ where *n* describes the filter length and *D* the vessel diameter.^18^ A one-dimensional median filter was applied to neighboring shear rates along the luminal border for further smoothing. To accommodate the low resolution with which the vessel wall was defined, the pixel coordinates of the wall were interpolated and the vessel wall was parameterized.

Characterizing the oscillatory near-wall flow, the oscillatory shear index (OSI_*xz*_) is defined

$${\text{OSI}}_{xz} = \frac{1}{2}\left( {1 - \frac{{\frac{1}{T}\left| {\mathop \smallint \nolimits_{0}^{t} \tau_{{{\text{w}},xz}} {\text{d}}t} \right|}}{{\frac{1}{T}\mathop \smallint \nolimits_{0}^{t} \left| {\tau_{{{\text{w}},xz}} } \right|{\text{d}}t}}} \right) = \frac{1}{2}\left( {1 - \frac{{\left|{{\overset{\lower0.5em\hbox{$\smash{\scriptscriptstyle\rightharpoonup}$}}{\tau}}_{{{\text{mean}},xz}} } \right|}}{{{\text{TAWSS}}_{xz} }}} \right) ,$$

where *t* is time, *T* the cardiac cycle, *τ*_w,*xz*_ the instantaneous WSS vector and TAWSS_*xz*_ the time average WSS in the imaging plane. Note that OSI_*xz*_ is a measure of oscillation and differs from the regularly formulated OSI: WSS components due to out-of-plane velocities are not included. The same is true for TAWSS_*xz*_. Nevertheless, high OSI remains associated with a low time average WSS, as with the conventionally-defined index.

## Results

### Ultrasound Simulation in an Idealized Geometry

**Figure 3:**
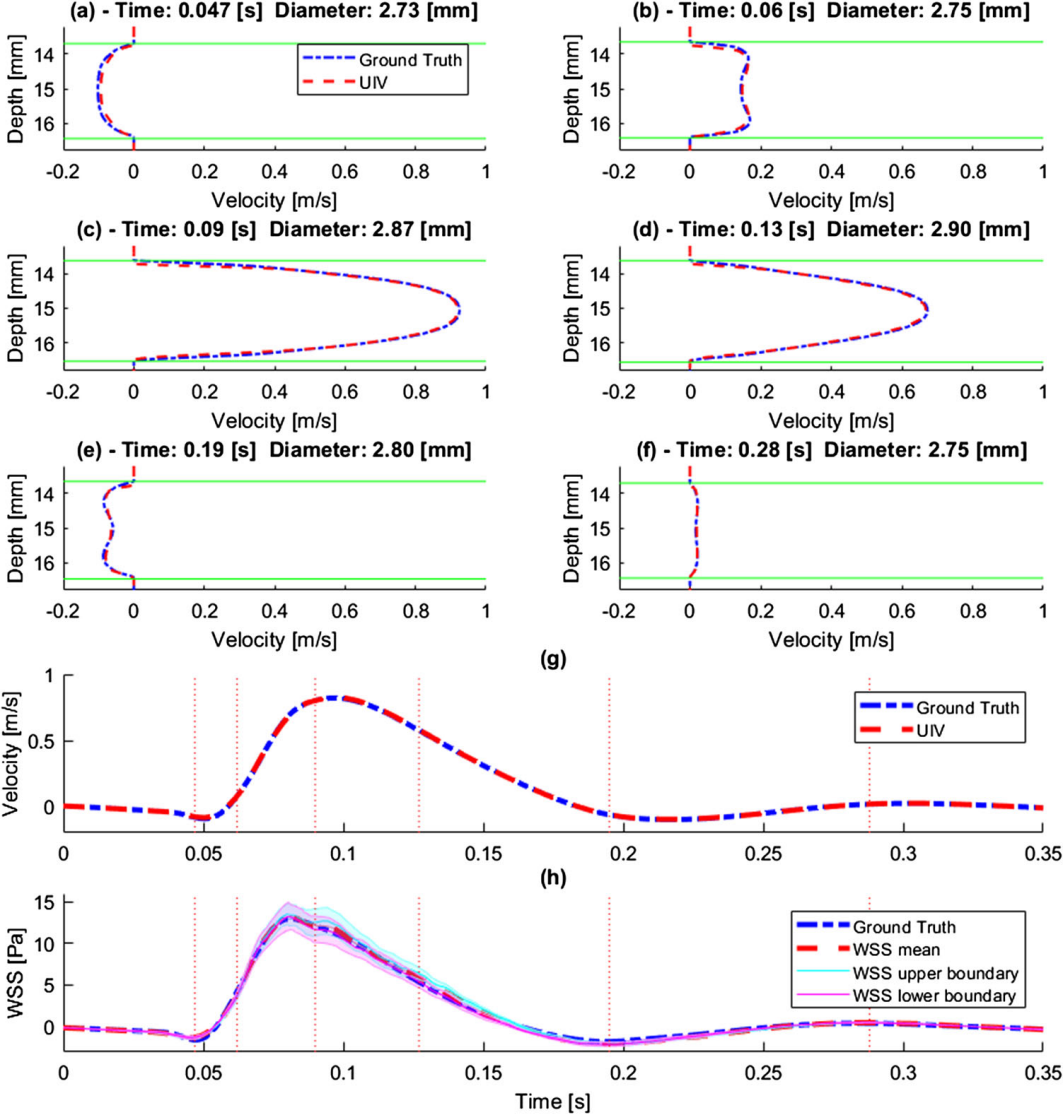
(a–f) Simulated ultrasound measurements of velocity profiles (and the corresponding ground truth) and vessel wall location at different points in the cardiac cycle in a vessel with moving boundaries. (g) Simulated measurements and ground truth velocity waveform, averaged over the whole image region. (h) WSS waveform on the upper or lower boundary, and the mean of WSS on both boundaries, calculated from the velocity profiles. Shaded area illustrates the standard deviation in the ROI. Vertical lines in panels (g) and (h) indicate the times for which panels (a–f) were derived.

Figure 3 illustrates the flow computed for a cylindrical segment of rabbit thoracic aorta with a realistic velocity waveform and moving walls, and for comparison also shows the simulated ultrasound measurements of this ground truth. Illustrations (a–f) show the velocity profile comparison at different points of the cardiac cycle along a central section of the image and the position of the segmented wall (green) with its corresponding diameter. Waveforms (g–h) illustrate the average velocity and WSS for all points of the image.

The normalized mean error over a cardiac cycle between the ground truth and the simulation of the measured average waveforms was 0.34% and 1.69% for velocity and WSS, respectively. In a point-by-point comparison, the mean error and standard deviation for velocity magnitude and flow direction were 5.65 ± 4.61% and 5.09 ± 15.09°, respectively. The excellent agreement between ground truth velocity profiles and the corresponding simulations of UIV measurements is apparent in Figs. 3a–3f. Furthermore, the simulated tracking of the vessel-wall boundary was accurate throughout the cardiac cycle, with average DSC = 0.99 and MAD = 24 *μ*m or ~ 1px.

### Vessel-Wall Segmentation

**Figure 4:**
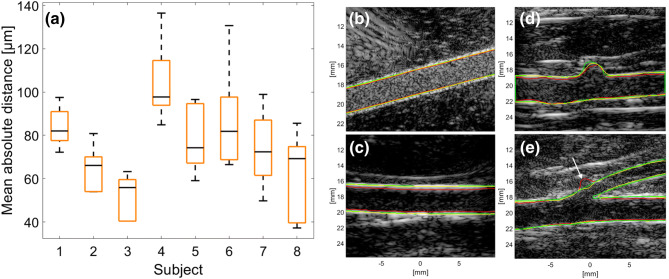
(a) Mean absolute distance between ground truth wall and segmented wall for 8 *in vivo* cases, averaged for 5 time points. The mean diameter of a rabbit abdominal aorta was 2.8 mm. (b–e) Examples of the wall tracking for ground truth (green), sparse field method (red) and sparse field method + directional peak fitting (yellow). Tracking was performed for simulations (b), the descending thoracic aorta (c), the abdominal aorta around the renal branch mouth (d) and the suprarenal abdominal aorta and celiac artery (e). The anomaly arrowed in (e) is discussed in the text.

Figure 4a summarizes the mean absolute distance between ground truth and automated vessel-wall tracking for eight rabbit aortas *in vivo*. Ground truth was determined by manual segmentation, repeated three times by an experienced user (KR). Vessel wall position was compared at five randomly picked points in the cardiac cycle. The overall (eight cases) mean diameter of the rabbit abdominal aorta was 2.8 mm and the maximum MADW was 137 *μ*m. The median MADW for all eight cases was below 97 *μ*m with a maximum standard deviation of 19.5 *μ*m. In all cases the Dice similarity coefficient (DSC) was > 0.97, suggesting very similar shapes.

Examples of ground truth and vessel segmentation from a simulation and measurements at three different locations in the vascular tree are illustrated in Figs. 4b–4e. In all cases, there is a gap between the SFM-derived mask and the ground truth. In the SFM + DPF final mask this gap is closed and the mask aligns with the bright wall. In the simulation (Fig. 4b), the walls of the vessel are clearly visible and well defined. *In vivo* the signal from the upper boundary bulges into the lumen (e.g., Fig. 4c); the vessel wall boundary cannot be clearly distinguished. On the side, the vessel wall boundary echoes spread just as widely, but outside the lumen area. Figure 4d illustrates the region around the left and right renal arteries. Unlike Figs. 4b and 4c, the wall is not straight. Figure 4e illustrates an example of the suprarenal abdominal aorta with two generations of branching, from the aorta to the celiac artery and thence to the gastric artery. The transition from the aorta to the celiac artery is well defined. However, the walls of the branch lumen and the protruding lip of the flow divider are hardly visible. In addition, the circular shape of the gastric artery (Fig. 4e, white arrow) suggests steeply out-of-plane alignment of the remaining conduit artery; the larger branch artery could be extracted accurately but the protruding lip of the flow divider and the smaller conduit artery could not.

### Measuring Flow and Calculating WSS in a Real Arterial Segment

**Figure 5:**
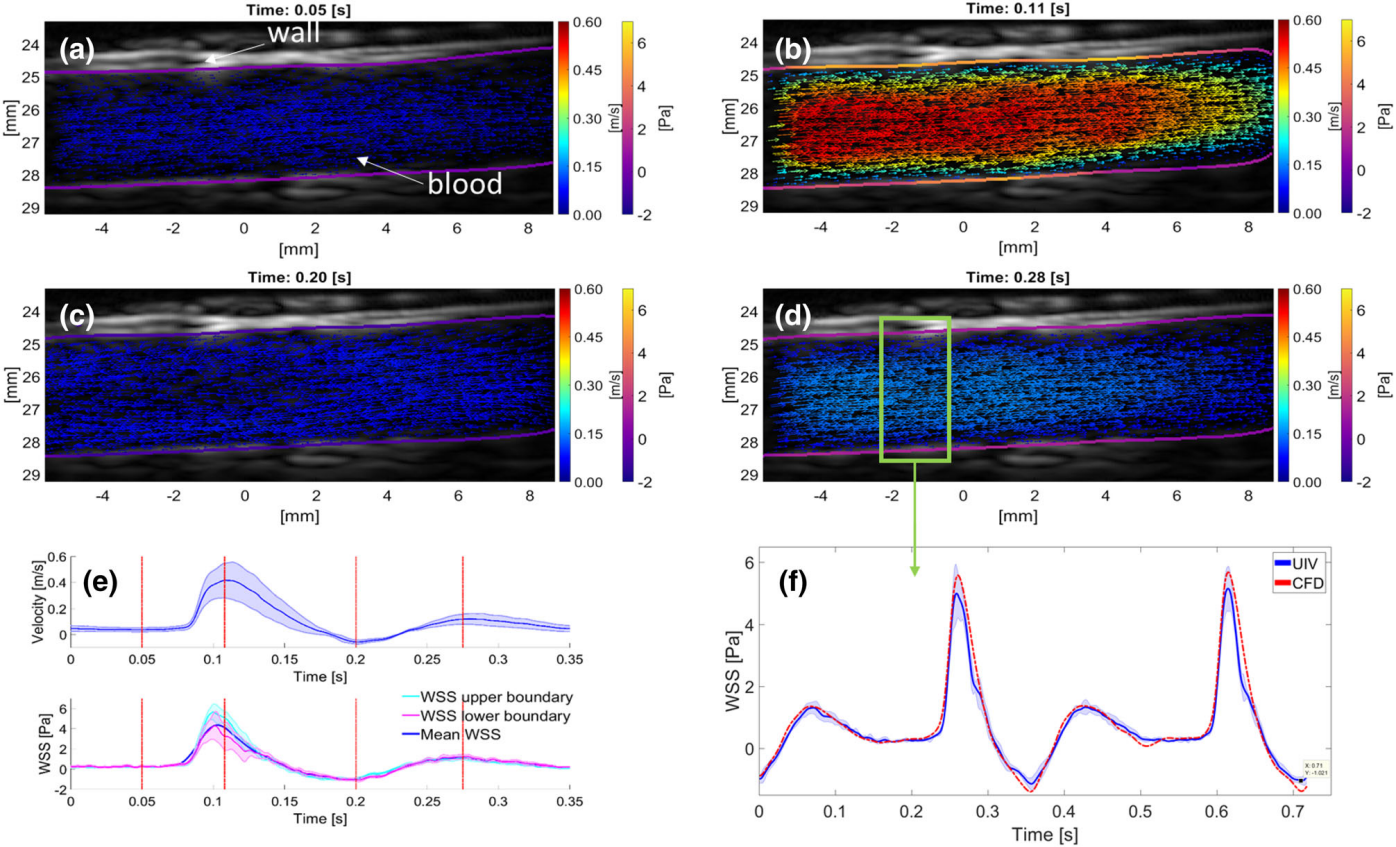
(a–d) UIV obtained velocity and wall shear stress maps in the abdominal aorta of a New Zealand White Rabbit at different points in the cardiac cycle. Forward flow from left to right. Color coding of vectors indicates speed and color coding of the luminal boundary indicates WSS. (e) Average waveforms from a single cardiac cycle. The point in time of each flow and WSS plot (a–d) is marked on the waveform plots. The shading in the velocity waveform plot represents the standard deviation over the whole image region. Wall shear stress measurements are illustrated for the upper and lower boundaries. Shaded area illustrates the standard deviation in the ROI. (f) Comparison of WSS waveforms between the *in vivo* acquisition (UIV) and animal matching simulation (CFD). Boundary conditions (wall displacement and velocity profile) were extracted from the region marked by a green box in (d) and hence are different from (e).

Figures 5a–5e illustrate the velocity vector field measured in a straight, unbranched segment of the descending thoracic aorta of a NZW rabbit at different points during the cardiac cycle, and the calculated WSS. The mean velocity and WSS waveforms are also shown. At 0.05 s into the cardiac cycle (Fig. 5a), blood moved purely in the forward direction (left to right) and with a low mean velocity and low average WSS = 0.4 Pa. At peak systole (0.1 s; Fig. 5b), peak velocity was $v = 0.5\,{\text{m}}/{\text{s}}$ and mean WSS = 4.4 Pa. At 0.2 s (Fig. 5c), following systole, a large backward flow and a negative WSS = − 1 Pa were observed. At 0.28 s, in the later stages of diastole (Fig. 5d), the blood accelerated forward again with a mean WSS = 1.2 Pa.

During systole, the average WSS was smaller on the lower boundary than the upper boundary; for the remaining part of the cardiac cycle, WSS was the same for both locations (Fig. 5f). The measured TAWSS_*xz*_ = 0.54 Pa was higher than the analytically derived Poiseuille TAWSS_HP_ = 0.46 Pa. Figure 5f compares measured and simulated WSS waveforms for the ROI illustrated by the green box in Fig. 5d. Wall displacement and velocity waveforms matched the *in vivo* acquisition. The Pearson correlation coefficient PCC_wss_ = 0.99 demonstrates close agreement between the measured and simulated WSS waveforms. (In the simulation, the vessel was assumed to be cylindrical.) The measurements gave a stair case decrease in WSS while in simulations the decrease was steady. The root mean square error and the normalized root mean square error were 0.29 Pa and 5.16%, respectively. The CFD-derived maximum WSS_CFD_ = 5.5 Pa was higher than the WSS_UIV_ = 5 Pa. During backward flow the minimum measured WSS_UIV_ = 1.13 Pa was higher than the CFD-derived WSS_CFD_ = 1.37 Pa.

### Estimating Flow and WSS Around the Origins of the Renal Arteries

**Figure 6:**
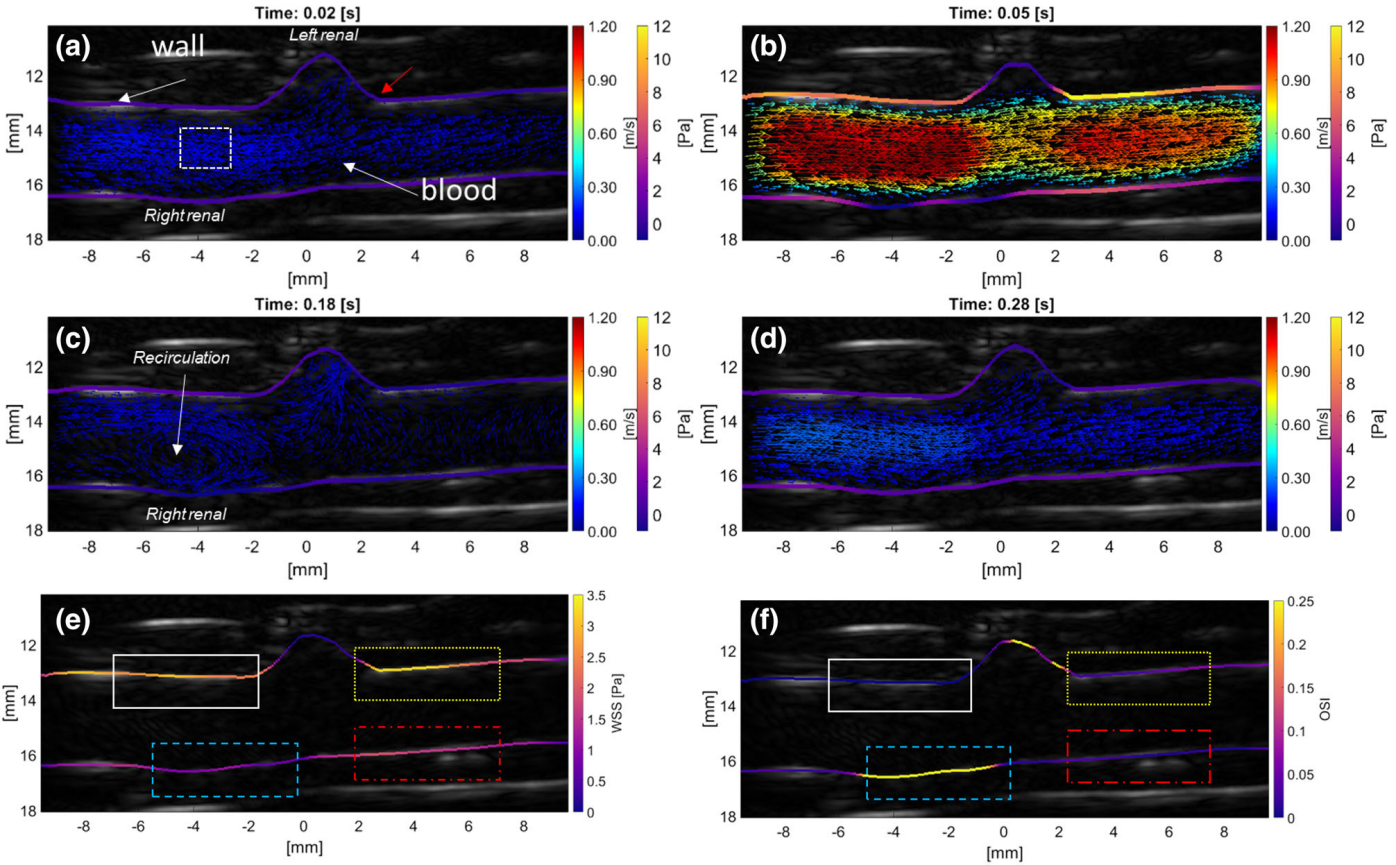
(a–d) UIV obtained velocity and wall shear stress maps around the renal artery branch mouths in the aorta of a New Zealand White Rabbit at different points in the cardiac cycle. Forward flow from left to right. Color coding of vectors indicates speed and color coding of the luminal boundary indicates WSS. Red arrow in (a) marks the flow divider. (e) TAWSS_*xz*_ and (f) OSI_*xz*_ averaged over three cardiac cycles. Putative atheroprone and atheroprotected sites around the branch mouth are highlighted in colored ROIs. High TAWSS_*xz*_ (white -, yellow ·) corresponds spatially with a low OSI_*xz*_. High OSI_*xz*_ (blue - -) can be observed downstream of the right renal branch and elevated TAWSS_*xz*_ levels (red -·-) are found opposite the left renal branch.

Figure 6 illustrates velocity and WSS maps around the origin in the aorta of the right and left renal arteries. At 0.02 s into the cardiac cycle (Fig. 6a), the flow was purely forward with a relatively low mean velocity of $v = 0.2\,{\text{m}}/{\text{s}}$ and average WSS = 1 Pa. During peak systole, at 0.05 s (Fig. 6b), the mean velocity and WSS reached their maxima of $v = 1\,{\text{m}}/{\text{s}}$ and WSS = 7 Pa. High velocities and WSS were found both upstream and downstream of the left renal branch. At 0.18 s (Fig. 6c), a large recirculation region developed around the right renal branch mouth. The net flow appeared to be into the conduit arteries and no forward flow in the aorta was observed, with mean WSS = 0 Pa. During diastole, at 0.28 s (Fig. 6d), the flow of blood in the aorta accelerated in the forward direction again, with mean $v = 0.17\,{\text{m}}/{\text{s}}$ and WSS = 0.7 Pa.

The TAWSS_*xz*_ and OSI_*xz*_ are illustrated in Figs. 6e and 6f. Regions (delineated white -, yellow ·) around the left renal branch ostium showed a high shear stress throughout the cardiac cycle. The region around the right renal artery (blue) had the lowest TAWSS_*xz*_. Downstream of the right renal artery (red -·-) the TAWSS_*xz*_ increased again. Low TAWSS_*xz*_ corresponded spatially with a high ${\text{OSI}}_{xz}$ and *vice versa*.

### Estimating Flow and WSS Around the Origin of the Celiac Artery

**Figure 7:**
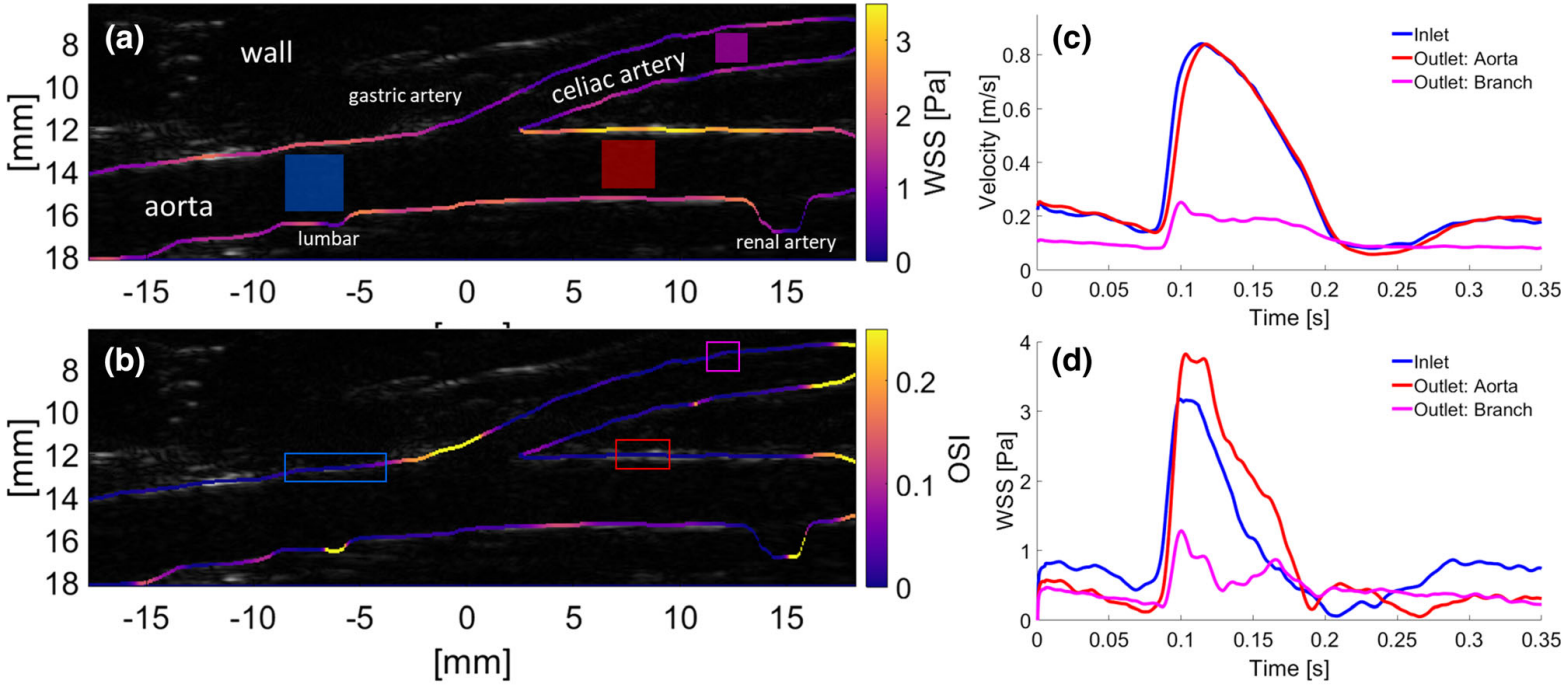
(a) TAWSS_*xz*_ and (b) OSI_*xz*_ averaged over two cardiac cycles around the origin of the celiac bifurcation. (c) velocity and (d) WSS waveform for different positions (colored ROIs) in the aorta and its conduit artery. The color of the boxes corresponds to colors in the plots of waveform. Color coding of the lumnal boundary indicates WSS metric values.

Figure 7 illustrates the TAWSS_*xz*_ and OSI_*xz*_ in the aorta around the origin of the celiac artery, and UIV-derived velocity and WSS waveforms at selected sites in the image. The wavy shape of the lower wall indicates several smaller branches, such as a lumbar vessel and a renal artery, leaving the aorta.

The maximum TAWSS_*xz*_ = 3.3 Pa was observed downstream of the branch; it was similar both in magnitude and location to the maximum at the renal ostium. However the wall upstream of the branch experienced a substantially lower TAWSS_*xz*_ = 2 Pa than at the renal ostium and had the highest OSI_*xz*_ = 0.3. As seen around the renal ateries, there was oscillatory flow opposite the branch, where OSI_*xz*_ = 0.14.

Concerning instantaneous values, blood flow velocity and WSS in the celiac artery during systole were substantially lower than in the aorta. WSS in the aorta was highest upstream of the branch during diastole and highest downstream during systole, despite the fact that the velocity waveforms at the two locations were very similar. This presumably reflects differences in velocity profile or inconsistent and uncorrelated errors in vessel tracking throughout the cardiac cycle.

## Discussion

Determining WSS accurately poses two key challenges: to measure slow near-wall flow unambiguously and to find the exact location of the vessel wall. Results from earlier studies give confidence in the ability of UIV to provide accurate estimates. In one study, *in vitro* echo PIV performed similarly well to optical PIV, with errors in 1-D WSS measurement for the two methods of 8 and 6.5%, respectively, using a third-degree polynomial curve fit.^14^ Another study obtained 1-D WSS measurements within 5% of the reference value *in vitro*; WSS measurement *in vivo* was conceptually demonstrated.^25^ Two-dimensional shear rate measurements have been validated *in vitro* against peak velocities measured by Doppler, UIV deviating by a maximum of 6.6%.^19^ A high correlation between echo PIV and optical PIV for WSS measurements has been observed in a realistic carotid bifurcation model using 2-D velocity vectors.^35^ And clinical studies showed high repeatability^9^ and low absolute differences of 17.0 ± 15.3% in WSS measurement by UIV compared to phase contrast MRI.^34^ A clinical comparison study of Doppler and UIV WSS measurement has underlined the ability of echo PIV to measure WSS with high accuracy.^8^ In these studies, WSS measurements were mainly performed on single vessels parallel to the lateral direction of the ultrasound transducer.

In the present study, we demonstrated wall tracking, velocity and WSS measurements using high-frame-rate, contrast-enhanced, incoherent ensemble-correlation UIV *in vivo* regardless of the number of vessels, beam angle, vessel curvature or branching. The measured flow field was fully two-dimensional and WSS values were obtained from velocity profiles that were locally perpendicular to the wall.

The ability to measure WSS was first validated in a realistic *in silico* simulation of flow and UIV in the thoracic aorta tilted at 15°, with flow and geometric boundary conditions based on real data. Both 2D velocities and WSS waveforms were accurately measured in the simulations, with a normalized mean error of 0.34 and 1.69%, respectively. More interesting was the ability to measure complex flow patterns: a low mean velocity bias (5.65%) and angle error (5.09°) were achieved, demonstrating high fidelity to the true flow. Similarly, a MADW = 24 *μ*m illustrates excellent tracking of the vessel boundary. Note, however, that the fully developed speckle pattern, lack of nonlinear response, uniform scatter size, two-dimensional scatter position and rigid motion in the simulation might not fully mimic *in vivo* conditions.

In different segments of the thoracic and abdominal aorta *in vivo* the lumen shape was well estimated with a mean DSC > 97% and a median MADW < 97 *μ*m. Nevertheless, a 97 *μ*m offset in wall localization could potentially lead to significant inaccuracies. Previous studies have shown that while a deviation of 80 *μ*m leads to an error in WSS of up to only 10%,^26^ a deviation of 200 *μ*m can give errors up to 60%.^18^ The seriousness of the error was therefore further investigated by comparing the measured ${\text{TAWSS}}_{xz} = 0.54\,{\text{Pa}}$ (Fig. 5) to the value computed from Poiseuille theory in the same geometry: ${\text{TAWSS}}_{\text{HP}} = 0.46\,{\text{Pa}}$. This difference represents a relatively modest deviation of 17%. Here, the measured WSS was compared to the analytical solution due to the lack of a ground truth.

Indeed, the average waveform for both velocity and WSS in Fig. 5 not only seem plausible but are highly correlated with the simulation results with a PCC_wss_ = 0.99. The root mean square error and the normalized root mean square error are 0.29 Pa and 5.16% respectively and WSS magnitudes are in a plausible range given the velocities. In addition, the maximum measured ${\text{TAWSS}}_{\text{xz}} = 4.4\,{\text{Pa}}$ over the whole image region is in agreement with previous studies in the abdominal aorta of NZW rabbits using real time measurements from an intravascular catheter: WSS = 5.12 Pa.^1^ Discrepancies between the second CFD simulation and the UIV experiment could be due to assumptions made in the simulation: a circular geometry with constant diameter lengthwise, fully-developed flow and rigid motion were assumed. In the experiment the vessel tapered, the walls bent and non-rigid motion probably occurred.

Comparing the distribution of WSS we obtained in regions of branching (Figs. 6 and 7) with CFD-derived maps of WSS, the agreement is good: TAWSS can be elevated both upstream and downstream of a branch and the magnitude of the measured ${\text{TAWSS}}_{xz}$ and OSI_*xz*_ are in the right range.^12^ Fluctuations in WSS around branch points and in areas of curvature are thought to explain the patchy distribution of atherosclerosis at such sites but there is disagreement about which aspects of the complex spatiotemporal WSS behavior are responsible. The development of methods for measuring near-wall blood flow is key to increasing our understanding and hence treatment of this disease.

Finally, we note that the biggest limitation of assessing WSS in the present study, and in all similar studies, is the 2D nature of the measurements. If velocities are measured in a plane that is not in alignment with the predominant flow direction, which is usually but not necessarily the long axis of the vessel, then WSS will be underestimated, and if the vessel is non-planar there may be more underestimation in some parts of the image than in others. The wall locations will also not represent a true diameter. Where there is planar geometry and accurate alignment, but out-of-plane flow, then TAWSS_*xz*_ and OSI_*xz*_ may be measured correctly but might still not be useful. Especially in regions of branching, where out of plane flow is expected, interpretation of the measured WSS is only qualitative. Furthermore, inaccuracies in determining the wall location in a vessel are uncorrelated and might lead to different degrees of error of WSS measurement along the image plane. Future work should aim to derive WSS from fully 3D measurements of flow by acquiring 4D data,^5^,^31^ by measuring multiple planes^37^ or by using speckle decorrelation methods.^36^

Despite its limitations we demonstrated that 2D UIV can be used to map ${\text{TAWSS}}_{xz}$ and OSI_*xz*_ in the abdominal aorta of NZW rabbits. Hence 2D UIV can be used to study the formation and progression of cardiovascular disease.
